# Effects of Dexmedetomidine Treatment After Cerebral Ischemia/Reperfusion on Apoptosis and Oxidative Stress: A Rat Model

**DOI:** 10.3390/life16020325

**Published:** 2026-02-13

**Authors:** Mahir Kuyumcu, Eda Yıldızhan

**Affiliations:** 1 Department of Anesthesia, Faculty of Medicine, Dicle University, Diyarbakır 21280, Turkey; 2 Department of Histology and Embryology, Faculty of Medicine, Recep Tayyip Erdoğan University, Rize 53100, Turkey; edayildizhan21@gmail.com

**Keywords:** cerebral ischemia–reperfusion, dexmedetomidine, oxidative stress, apoptosis, Bax/Bcl-2/APAF-1, neuroprotection, bioinformatics, molecular docking

## Abstract

Objectives: Cerebral ischemia/reperfusion (IR) injury is characterized by excessive oxidative stress and activation of apoptotic pathways, which play a central role in neuronal loss and poor neurological outcomes. Modulation of these mechanisms represents a clinically relevant strategy for neuroprotection. This study aimed to investigate the neuroprotective effects of dexmedetomidine (Dex) on oxidative stress, apoptotic signaling, and neuronal integrity in an experimental rat model of cerebral IR injury. Materials and Methods: Female Wistar rats were assigned to control, IR, and IR+Dex groups. Transient cerebral ischemia was induced for 45 min followed by 2 h of reperfusion. Oxidative stress was evaluated using serum antioxidant enzyme activities (superoxide dismutase [SOD], catalase [CAT], glutathione peroxidase [GSH-Px]), total oxidant and antioxidant status (TOS, TAS), and lipid peroxidation levels (malondialdehyde [MDA]). Apoptotic signaling was assessed by histopathological examination, transmission electron microscopy, and immunohistochemical analysis of B-cell lymphoma 2 (Bcl-2), Bcl-2-associated X protein (Bax) and apoptotic peptidase activating factor-1 (APAF-1) expression, quantitatively evaluated using QuPath with statistical comparison between groups. Bioinformatic network analysis and molecular docking were performed to explore predicted interactions between Dex and apoptosis-related proteins. Results: Cerebral IR induced a marked oxidative imbalance, characterized by reduced antioxidant enzyme activities and increased lipid peroxidation. Dex treatment partially improved antioxidant capacity and reduced oxidative stress parameters. Histopathological and ultrastructural analyses demonstrated severe neuronal degeneration following IR, whereas Dex-treated rats exhibited attenuated neuronal damage and improved ultrastructural preservation. Immunohistochemical analysis showed increased Bax and APAF-1 expression and reduced Bcl-2 expression after IR; these alterations were significantly modulated toward control levels in the IR+Dex group. Bioinformatic analysis identified apoptosis-related pathways, including apoptosis, p53 signaling, and necroptosis, as significantly enriched, while molecular docking suggested stable predicted interactions between Dex and key apoptotic regulators. Conclusions: In this experimental rat cerebral IR model, Dex exerted partial but significant neuroprotective effects by attenuating oxidative stress, modulating apoptotic marker expression, and preserving neuronal morphology. These findings support the potential role of Dex as a neuroprotective agent in ischemia-related brain injury, warranting further translational investigation.

## 1. Introduction

Cerebral ischemia/reperfusion (IR) injury arises when the restoration of blood flow after an ischemic insult paradoxically accelerates neuronal damage, primarily through excessive generation of reactive oxygen species (ROS), activation of inflammatory cascades, and disruption of cellular homeostasis [1]. Cerebral ischemia/reperfusion leads to ionic imbalances, particularly involving calcium (Ca^2+^), sodium (Na^+^), and potassium (K^+^), which contribute to mitochondrial dysfunction and neuronal injury [2]. Experimental models demonstrate that cerebral IR triggers lipid peroxidation, blood–brain barrier dysfunction, neuronal swelling, and extensive cell death through ROS-mediated mitochondrial injury, cytochrome-c release, and caspase activation [2,3].

The mechanisms underlying cerebral IR remain incompletely understood; however, accumulating evidence highlights oxidative stress, neuroinflammation, and apoptotic signaling as central mediators of injury [4]. Excessive ROS production during reperfusion overwhelms endogenous antioxidant systems, leading to increased malondialdehyde (MDA) levels and reduced activity of superoxide dismutase (SOD), catalase (CAT), and glutathione peroxidase (GSH-Px) [5]. Concurrently, pro-inflammatory cytokines such as tumor necrosis factor (TNF)-α and interleukin (IL)-6 rise rapidly following reperfusion, exacerbating neuronal degeneration, while microglial activation contributes to secondary injury [6]. These processes converge on mitochondrial apoptotic pathways involving Bax/Bcl-2 imbalance, release of cytochrome-c, and activation of caspase-3 and APAF-1, collectively promoting widespread neuronal death [7].

Dexmedetomidine (Dex) is a clinically approved, highly selective α2-adrenergic receptor agonist widely used for sedation and analgesia in intensive care and perioperative settings that has gained substantial attention not only for its anesthetic and sedative properties but also for its promising organ-protective effects across various IR models [8]. Numerous studies indicate that Dex reduces oxidative stress, inhibits inflammatory signaling, and attenuates apoptotic injury in cerebral, cardiac, hepatic, renal, and intestinal IR settings [9,10]. Experimental work in cerebral ischemia models shows that Dex decreases neuronal apoptosis, preserves hippocampal integrity, and modulates microglial activation by downregulating Bax, caspase-3, Nox2, and pro-inflammatory cytokines [11]. Mechanistically, dexmedetomidine exerts its neuroprotective effects through the coordinated activation of multiple intracellular survival pathways, including PI3K/Akt/mTOR, HIF-1α/p53, JAK/STAT3, MAPK, and thioredoxin-1 (Trx-1)–dependent antioxidative signaling [9,10]. In particular, activation of the PI3K/Akt axis leads to downstream phosphorylation of mammalian target of rapamycin (mTOR), a central regulator of cellular metabolism, protein synthesis, and survival, thereby promoting neuronal resistance to ischemia-induced oxidative stress and apoptosis [12,13,14]. Through modulation of these interconnected pathways, dexmedetomidine stabilizes mitochondrial function, limits endoplasmic reticulum stress, and suppresses pro-apoptotic signaling cascades during cerebral ischemia/reperfusion injury [15].

Unlike previous dexmedetomidine studies that primarily focused on isolated biochemical or histological endpoints [16,17,18], the present study adopts an integrative experimental design combining biochemical, histopathological, immunohistochemical, ultrastructural, and in silico analyses within the same cerebral ischemia/reperfusion model. Given the complex interplay between oxidative stress, neuroinflammation, and apoptosis in the ischemic brain [19,20,21], this approach enables the simultaneous evaluation of systemic oxidative stress, neuronal morphology, apoptosis-related protein expression, and predicted molecular interaction networks. By linking tissue-level findings with bioinformatic pathway enrichment and docking-based predictions, the study provides a more comprehensive mechanistic framework for understanding dexmedetomidine-mediated neuroprotection.

The present study aims to comprehensively investigate the neuroprotective effects of Dex in a rat cerebral IR model, with a specific focus on oxidative stress regulation and apoptotic pathway modulation. By integrating in vivo biochemical markers, neuronal morphology, immunohistochemical expression of Bax, Bcl-2, and APAF-1, and in silico interaction network analyses, this study seeks to elucidate the multifaceted mechanisms through which Dex mitigates cerebral IR-induced neuronal injury.

## 2. Materials and Methods

### 2.1. Ethics Committee Approval

All experimental procedures were conducted in strict accordance with the principles outlined in the Guide for the Care and Use of Laboratory Animals. Ethical approval for the study was granted by the Dicle University Local Animal Experiments Ethics Committee (DÜHADEK) under protocol number 2025/01, dated 27 February 2025. All efforts were made to minimize the number of animals used and to reduce suffering during the experimental protocol.

### 2.2. Cerebral IR Model

Prior to surgical intervention, general anesthesia was induced in all rats via intraperitoneal administration of 50 mg/kg ketamine hydrochloride and 10 mg/kg xylazine hydrochloride, consistent with previously validated cerebral ischemia protocol [22]. The animals were positioned supine on a temperature-controlled operating platform. Following a midline cervical incision, the external carotid artery (ECA) and internal carotid artery (ICA) were carefully isolated using blunt dissection under an operating microscope. Transient focal cerebral ischemia was induced by applying a temporary microvascular arterial clamp to the ICA. A standardized ischemia period of 45 min was maintained to ensure reproducibility of the model, after which the clamp was gently removed to allow reperfusion for 2 h, consistent with established middle cerebral artery occlusion (MCAO) reperfusion parameters [23]. Throughout IR, body temperature was continuously monitored via a rectal probe and maintained between 37 °C and 37.5 °C with an external warming system to prevent hypothermia-related confounders. To avoid dehydration and maintain hemodynamic stability, all animals received subcutaneous administration of isotonic saline at a rate of 3 mL/kg/h during the surgical procedure.

### 2.3. Administration of Dex

Dex was administered intraperitoneally at a dose of 100 μg/kg per application. The dose and timing of Dex administration were selected based on previously published experimental studies demonstrating neuroprotective efficacy in cerebral and systemic ischemia/reperfusion models [2,22,23]. The same administration protocol was applied before ischemia to ensure adequate systemic distribution during both the ischemic and reperfusion phases.

### 2.4. Estrous Cycle Monitoring

To ensure hormonal consistency among animals, estrous cycle staging was performed using the vaginal smear cytology technique. Vaginal epithelial cells were collected by gentle lavage, air-dried, stained, and examined microscopically. Only rats identified as being in the same estrous phase were included in the experimental groups to minimize hormonal variability.

### 2.5. Formation of Experimental Groups

The study utilized female Wistar Albino rats, aged 8–10 weeks and weighing 200–250 g. Animals were housed under standard laboratory conditions in stainless-steel cages at 22 ± 2 °C, maintained on a 12 h light/12 h dark photoperiod, and provided with standard chow and tap water ad libitum. In alignment with the study objectives, rats were randomly assigned to four groups:

Control group (*n* = 7): Animals in this group underwent neither surgical intervention nor pharmacological treatment and served as the baseline reference.

IR group (*n* = 7): IR injury was induced under general anesthesia using ketamine hydrochloride (50 mg/kg, i.p.) and xylazine hydrochloride (10 mg/kg, i.p.). A 45 min ischemia period was followed by 2 h of reperfusion. Supplemental anesthesia was administered every 45 min to maintain adequate depth throughout the ischemic phase.

IR+Dex Group (*n* = 7): Rats received Dex (100 μg/kg, i.p.) prior to the induction of ischemia. Dex administration was continued during the 2 h reperfusion period following the initial 45 min ischemic insult, ensuring continuous pharmacological exposure throughout the reperfusion phase.

### 2.6. Biochemical Analyses

Blood samples were collected from the hearts of rats subjected to the experimental protocol, and the animals were euthanized. The collected blood samples were centrifuged at 3000 rpm for 8 min to separate the serum fraction, which was then prepared for analysis. Malondialdehyde (MDA, #MAK085, Merck KGaA, Darmstadt, Germany), Total Antioxidant Status (TAS, #NN21117A, Rel Assay Diagnostics, Gaziantep, Turkey), Total Oxidant Status (TOS, #NN211290, Rel Assay Diagnostics, Gaziantep, Turkey), Superoxide Dismutase (SOD, #MBS2707324, MyBioSource, San Diego, CA, USA), Catalase (CAT, #MBS726781, MyBioSource, San Diego, CA, USA) and Glutathione Peroxidase (GSH-Px, #354104, Merck KGaA, Burlington, MA, USA) were measured using rat-compatible Enzyme-Linked Immunosorbent Assay (ELISA) kits [24].

### 2.7. Histological Tissue Processing

Brain tissues were fixed in 10% formalin (Sigma Aldrich, St. Louis, MO, USA) for 48 h, then dehydrated using ethanol series of increasing concentrations. Subsequently, the tissues were processed using routine histological procedures, and 5 µm thick sections were prepared from paraffin blocks. The prepared sections were stained with Hematoxylin and Eosin (H&E) and examined under a Zeiss brand light microscope (Carl Zeiss, Baden, Germany).

### 2.8. Immunohistochemical Staining of Bax, Bcl-2, or APAF-1

Immunohistochemistry was performed to evaluate apoptotic protein expression (Bax, Bcl-2, APAF-1, catalog numbers: sc-7480; sc-65891; sc-13561; Santa Cruz Biotechnology Inc., Heidelberg, Germany; dilution: 1/100, respectively). Paraffin-embedded sections were deparaffinized in xylene, rehydrated through descending alcohol series, and exposed to heat-induced antigen retrieval using citrate buffer (pH 6.0) for 20 min. Endogenous peroxidase activity was quenched with 3% hydrogen peroxide for 10 min. Sections were blocked with normal serum and incubated overnight at 4 °C with primary antibodies against Bax, Bcl-2, or APAF-1. After washing, slides were incubated with biotinylated secondary antibodies followed by HRP-conjugated streptavidin. Visualization was performed using 3,3′-diaminobenzidine (DAB), and nuclei were counterstained with hematoxylin. Slides were mounted with mounting medium and imaged under Zeiss Imager A2 (Germany) bright-field microscopy [25]. To measure quantitatively expression protein markers, regions of interest were selected from anatomically comparable cerebral cortical areas across all animals, avoiding tissue edges and processing artifacts. For all markers, identical QuPath detection parameters were applied to ensure consistency. Quantitative values were averaged per animal prior to statistical analysis. For each animal, at least five non-overlapping high-power fields were analyzed, and mean values were used for statistical evaluation for QuPath analysis.

### 2.9. Network Construction and Pathway Analysis

To elucidate the potential molecular pathways through which Dex may mediate its neuroprotective effects, Kyoto Encyclopedia of Genes and Genomes (KEGG) pathway enrichment analysis was performed using the common targets obtained from the intersection of the proteins targeted by Dex and the apoptosis-related proteins investigated in this study (Bax, Bcl-2, and APAF-1). The target protein network of Dex was retrieved from the Search Tool for Interactions of Chemicals (STITCH) database, with the number of additional interactors set to 20 in both the first and second shells and a medium confidence score (≥0.4) [26]. The interaction networks of the apoptotic proteins were obtained from the Search Tool for the Retrieval of Interacting Genes (STRING) database, including 100 additional interactors, and visualized using Cytoscape software (version 3.10.3). The resulting networks were intersected to identify common proteins, which were subsequently subjected to KEGG pathway enrichment analysis using the ShinyGO platform (version 0.85.1) [27]. Statistical significance was defined as a false discovery rate (FDR) < 0.05, and the top 10 most significantly enriched KEGG pathways were ranked based on fold enrichment [28].

### 2.10. Ultrastructural Analysis

Cerebral cortical tissue samples were immediately collected following euthanasia and cut into 1 mm^3^ blocks. Specimens were fixed in 2.5% glutaraldehyde in 0.1 M phosphate buffer (pH 7.4) at 4 °C for 24 h. After primary fixation, tissues were washed in phosphate buffer and post-fixed in 1% osmium tetroxide for 1 h. Samples were then dehydrated through a graded ethanol series (30–100%) and cleared in propylene oxide. Following dehydration, the tissues were infiltrated and embedded in epoxy resin. Ultrathin sections (approximately 60–80 nm) were cut using an ultramicrotome and placed on copper grids. Sections were stained with uranyl acetate and lead citrate to enhance contrast. The ultrastructural examination was performed using a transmission electron microscope (e.g., JEOL or Hitachi model), operated at 80–120 kV. Digital micrographs were captured for analysis of nuclear morphology, chromatin pattern, cytoplasmic vacuolization, and mitochondrial integrity [29].

### 2.11. Molecular Docking Analysis

Molecular docking simulations were performed to evaluate the binding affinity between Dex and the apoptosis-related target proteins of interest, including Bax, Bcl-2, APAF-1, Caspase-3 (Casp3), Caspase-8 (Casp8), and Caspase-9 (Casp9), which were selected based on their central roles in intrinsic and extrinsic apoptotic pathways. The three-dimensional structures of these proteins were retrieved from the Protein Data Bank (PDB; https://www.rcsb.org/) using representative crystallographic entries with well-resolved ligand-binding or catalytic domains. The 3D structure of Dex (PubChem CID: 5311068) was downloaded in .sdf format from the PubChem database and converted to .pdbqt format using Open Babel (version 3.1.1). All protein and ligand structures were prepared for docking by removing crystallographic water molecules, adding polar hydrogens, and assigning Kollman charges using AutoDock Tools (version 1.5.7). Docking simulations were performed using AutoDock Vina (version 1.2.3) with an exhaustiveness parameter of 8. For each protein, the grid box was positioned to cover the biologically relevant binding region: the BH3 interaction groove for Bax and Bcl-2, the nucleotide-binding domain for APAF-1, and the active-site catalytic clefts for Casp3, Casp8, and Casp9. Grid dimensions were adjusted (typically 20 × 20 × 20 Å) to fully encompass the potential ligand-accessible pocket identified through cavity prediction. The most favorable docking pose for each protein was selected based on the lowest predicted binding energy (kcal/mol) and the presence of stabilizing interactions, including hydrogen bonds and hydrophobic contacts. Structural visualization and interaction analysis were conducted using Discovery Studio Visualizer (BIOVIA, San Diego, CA, USA) and PyMOL (version 2.5). Two-dimensional ligand–protein interaction maps were generated using LigPlot+ softwareversion 2.3 to illustrate key amino acid contacts contributing to complex stability.

### 2.12. Statistical Analysis

All data were analyzed using SPSS version 25.0 (IBM, Armonk, NY, USA). Normality of distribution was assessed with the Shapiro–Wilk test. Parametric data were expressed as mean ± standard deviation (SD) and analyzed using one-way ANOVA followed by Tukey’s post hoc test. A *p*-value < 0.05 was considered statistically significant. All analyses were performed by a biostatistician blinded to group allocation. A power analysis was performed prior to the study. The effect size was set at 0.70 according to Cohen’s effect size classification. With a statistical power of 0.75, the minimum required sample size was calculated to be 21.

## 3. Results

### 3.1. Cerebral IR Disrupts Redox Homeostasis While Dex Restores Antioxidant Balance

As shown in Table 1, cerebral ischemia/reperfusion (IR) resulted in a significant disturbance of systemic redox homeostasis compared with the control group. Antioxidant enzyme activities, including SOD, CAT, and GSH-Px, were significantly reduced in the IR group, while lipid peroxidation (MDA) and total oxidant status (TOS) were markedly increased, indicating enhanced oxidative stress. Dex administration partially attenuated these alterations. In the IR+Dex group, SOD, CAT, and GSH-Px levels were significantly higher than those observed in the IR group; however, these values did not fully return to control levels. Similarly, MDA and TOS levels were significantly reduced by Dex treatment compared with IR alone, although they remained elevated relative to controls. Total antioxidant status (TAS) was significantly decreased following IR and showed a partial recovery in the IR+Dex group, further supporting a moderate restoration of antioxidant capacity rather than complete normalization. Overall, these findings indicate that Dex partially improves oxidative balance following cerebral IR injury.

### 3.2. Dex Attenuates IR-Induced Neuronal Degeneration in Histopathological Assessment

**Figure 1:**
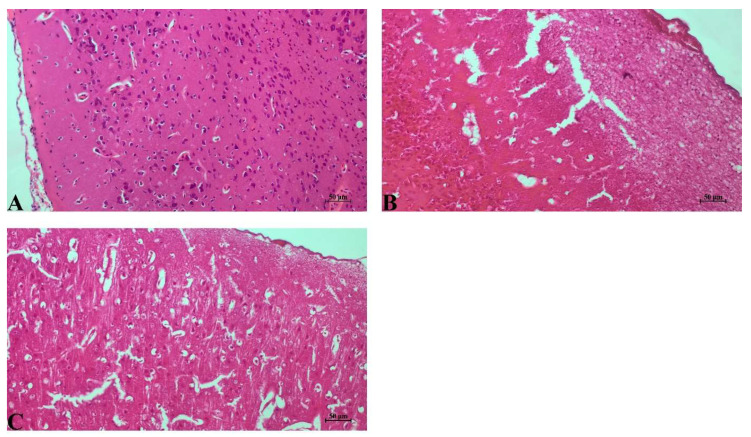
Representative H&E-stained coronal brain sections. (**A**) Control group showing normal neuronal morphology and preserved cytoarchitecture. (**B**) Cerebral IR group demonstrating neuronal pyknosis, cytoplasmic eosinophilia, vacuolar degeneration, and perivascular/perineuronal edema. (**C**) Cerebral IR+Dex group showing reduced degenerative changes and partial preservation of neuronal architecture. Staining: Hematoxylin and Eosin (H&E); Scale bar: 50 µm.

Control group: Cerebral cortical tissue exhibited normal histoarchitecture. Neurons displayed intact cell bodies, centrally located nuclei, and preserved Nissl substance. Neuropil organization was normal, with no evidence of edema, necrosis, vacuolization, or inflammatory cell infiltration. Vascular structures appeared intact without congestion (Figure 1A).

IR group: Marked histopathological injury was evident following IR. Neurons showed pronounced ischemic changes, including pyknotic nuclei, cytoplasmic eosinophilia, and shrunken cell bodies. Extensive vacuolization, spongiosis, and neuropil rarefaction were observed. Perivascular and intracellular edema were prominent, and disrupted tissue architecture indicated widespread necrotic injury (Figure 1B).

IR+Dex group: The treatment group exhibited substantial histological improvement compared with the cerebral IR group. Neuronal morphology was better preserved, with fewer pyknotic cells and reduced cytoplasmic eosinophilia. Tissue edema and vacuolization were noticeably diminished, and neuropil integrity was partially restored. Overall, neuronal degeneration and necrotic changes were markedly reduced in the IR+Dex group compared with the IR group.

### 3.3. Dex Reduces Bax Expression Elevated by Cerebral IR

**Figure 2:**
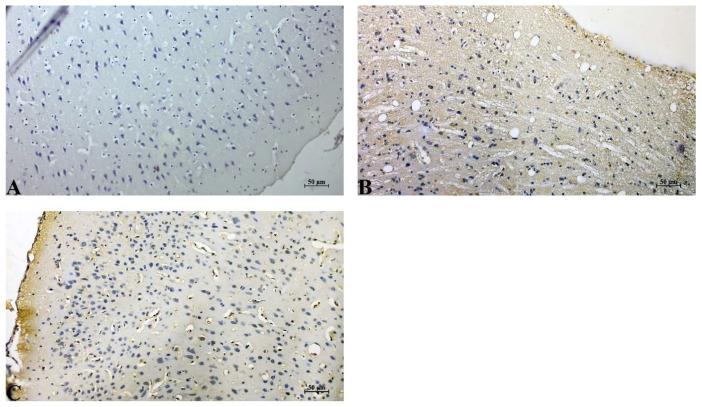
Immunohistochemical expression of Bax in cerebral cortical neurons. (**A**) Control group showing minimal Bax immunoreactivity. (**B**) Cerebral IR group exhibiting increased cytoplasmic Bax staining in neurons. (**C**) Cerebral IR+Dex group showing reduced Bax immunoreactivity compared with the IR group. Staining: DAB; Counterstain: Hematoxylin; Scale bar: 50 µm.

Control group: Cerebral cortical tissue exhibited minimal Bax immunoreactivity. Neurons showed faint or absent cytoplasmic staining, consistent with low baseline pro-apoptotic activity under physiological conditions. The neuropil and vascular compartments also lacked notable Bax expression (Figure 2A).

IR group: IR injury resulted in a marked increase in Bax expression. Strong cytoplasmic immunopositivity was observed in numerous neurons, particularly in the peri-infarct region. Staining intensity was diffuse and widespread, accompanied by increased cellular shrinkage and vacuolated neuropil (Figure 2B).

IR+Dex group: The treatment group displayed substantially reduced Bax immunoreactivity compared with the untreated cerebral IR group. Neuronal Bax staining was mild to moderate, with fewer strongly positive cells and overall lower intensity. Tissue morphology was better preserved, with diminished neuropil disruption. Strong cytoplasmic Bax immunoreactivity was observed in neurons of the IR group, whereas staining intensity and the number of positive cells were reduced in the IR+Dex group (Figure 2C).

### 3.4. Dex Restores IR-Suppressed Bcl-2 Expression Toward Baseline Levels

**Figure 3:**
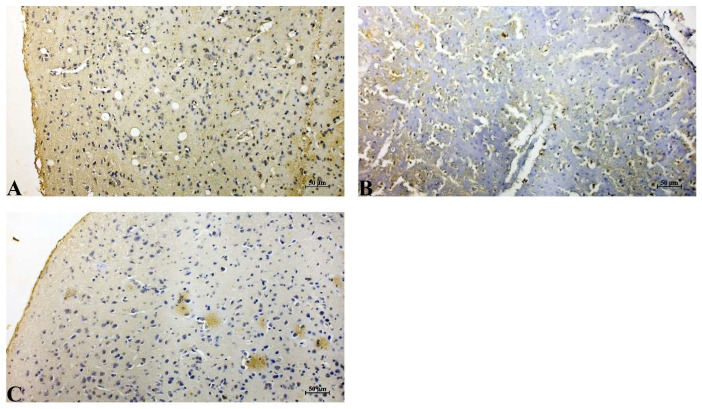
Immunohistochemical expression of Bcl-2 in cerebral cortical neurons. (**A**) Control group showing baseline Bcl-2 immunoreactivity. (**B**) Cerebral IR group demonstrating reduced Bcl-2 staining. (**C**) Cerebral IR+Dex group showing increased Bcl-2 immunoreactivity compared with the IR group. Staining: DAB; Counterstain: Hematoxylin; Scale bar: 50 µm.

Control group: Cerebral cortical tissue in the control group demonstrated baseline Bcl-2 immunopositivity, characterized by mild cytoplasmic staining in scattered neurons. The staining pattern was uniform and weak, consistent with normal physiological anti-apoptotic activity. No tissue disruption, edema, or background staining artifacts were observed (Figure 3A).

IR group: The IR group exhibited a marked reduction in Bcl-2 immunoreactivity. Neurons showed faint or nearly absent cytoplasmic staining, indicating a significant suppression of anti-apoptotic signaling following ischemic insult. This decrease in Bcl-2 expression was accompanied by widespread structural degeneration, including vacuolated neuropil, distorted neuronal morphology, and a pale background consistent with tissue necrosis (Figure 3B).

IR+Dex group: The treatment group displayed restoration of Bcl-2 expression, with moderate and clearly detectable cytoplasmic staining in numerous neurons. Staining intensity and distribution were visibly higher than in the untreated cerebral IR group, suggesting partial reactivation of anti-apoptotic pathways. Tissue morphology was comparatively preserved, with reduced vacuolization and improved neuronal integrity. These findings support the neuroprotective effect of the administered agent through enhancement of Bcl-2-mediated cell survival mechanisms (Figure 3C).

### 3.5. Dex Suppresses APAF-1 Upregulation Associated with IR-Driven Apoptosis

**Figure 4:**
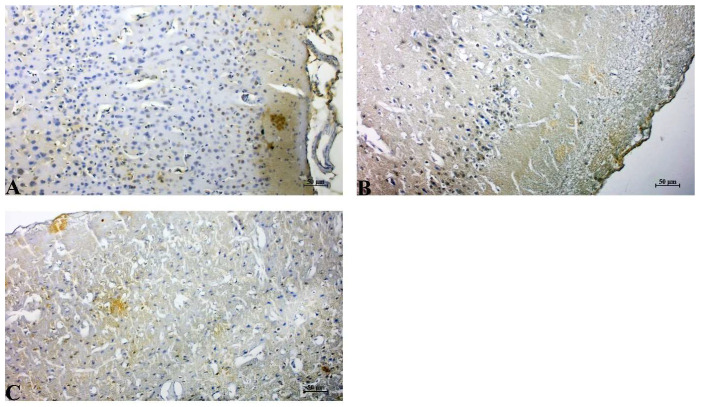
Immunohistochemical expression of APAF-1 in cerebral cortical neurons. (**A**) Control group showing minimal APAF-1 immunoreactivity. (**B**) Cerebral IR group exhibiting increased cytoplasmic APAF-1 staining. (**C**) Cerebral IR+Dex group showing reduced APAF-1 immunoreactivity compared with the IR group. Staining: DAB; Counterstain: Hematoxylin; Scale bar: 50 µm.

Control group: Cerebral cortical tissue in the control group exhibited minimal APAF-1 immunoreactivity. Neurons showed faint or absent cytoplasmic staining, consistent with physiologically low baseline expression of APAF-1. The neuropil appeared intact, and no pathological background staining was present (Figure 4A).

IR group: A pronounced increase in APAF-1 immunopositivity was observed following IR insult. Numerous neurons demonstrated strong cytoplasmic staining, accompanied by diffuse positivity throughout the damaged cortical region. The elevated APAF-1 signal correlated with apparent structural degeneration, including neuropil fragmentation, cellular shrinkage, and widespread vacuolization. This enhanced expression is consistent with increased apoptotic signaling following cerebral IR (Figure 4B).

IR+Dex group: The treatment group showed substantially reduced APAF-1 expression compared with the untreated cerebral IR group. Neuronal staining intensity was mild to moderate, and the number of strongly positive cells was notably lower. Tissue morphology was better preserved, with decreased vacuolization and improved cortical organization. The reduction in APAF-1 immunoreactivity suggests that the administered agent effectively attenuated cerebral IR-induced apoptotic signaling by modulating APAF-1–dependent apoptosome activation (Figure 4C).

### 3.6. Quantitative Image Analysis Confirms Dex-Mediated Modulation of Apoptotic Markers

QuPath-based quantitative analysis confirmed the qualitative immunohistochemical findings. Bax and APAF-1 expression were significantly upregulated in the cerebral IR group compared with the control group, whereas Bcl-2 expression was markedly reduced. In contrast, Dex treatment partially reversed these alterations. When Bax staining was quantified as the percentage of DAB-positive cells and H-score, the cerebral IR group exhibited a robust increase in Bax positivity relative to controls, consistent with enhanced apoptotic signaling. Dex administration significantly reduced Bax-positive cell percentages and H-scores compared with the cerebral IR group, although values remained slightly higher than in the control group. APAF-1 showed a similar pattern: cerebral IR markedly increased APAF-1-positive cells and H-scores. Dex treatment significantly decreased APAF-1 expression compared with the cerebral IR group, again approaching but not fully reaching control levels. In contrast, Bcl-2, an anti-apoptotic protein, was significantly decreased in the cerebral IR group compared with the control group. Dex administration significantly increased Bcl-2-positive cell percentages and H-scores relative to the cerebral IR group, restoring Bcl-2 expression toward baseline values. Overall, QuPath-based quantification demonstrated a clear shift in the Bax/Bcl-2/APAF-1 profile toward apoptosis in untreated cerebral IR, whereas Dex shifted this profile toward cell survival by reducing Bax and APAF-1 and increasing Bcl-2 expression. These quantitative findings are in line with the biochemical reduction in oxidative stress and the histopathological attenuation of neuronal damage observed in the Dex-treated group (Table 2).

Data are presented as mean ± SD. Immunohistochemical quantification was performed using QuPath software version 0.6.0 by analyzing at least five non-overlapping high-power fields per animal, and mean values were used for statistical analysis. Percentages of positive cells and H-scores were calculated for each marker. Statistical analysis was performed using one-way ANOVA followed by Tukey’s post hoc test. *p*-values indicate comparisons versus the IR group. A *p*-value < 0.05 was considered statistically significant.

### 3.7. Ultrastructural Evaluation Shows Improved Neuronal Integrity in IR+Dex Group

**Figure 5:**
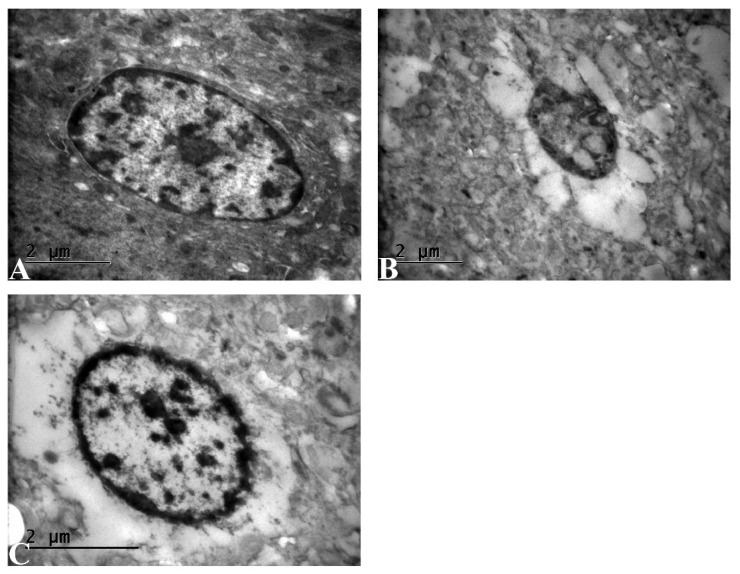
Transmission electron microscopy images of cerebral cortical neurons. (**A**) Control group showing preserved neuronal ultrastructure. (**B**) Cerebral IR group demonstrating marked ultrastructural degeneration, including chromatin condensation, cytoplasmic vacuolization, and mitochondrial swelling. (**C**) Cerebral IR+Dex group showing partial preservation of neuronal ultrastructure compared with the IR group. Scale bar: 2 µm.

Ultrastructural analysis of cerebral tissues is shown in Figure 5. Neurons in the control group exhibited normal ultrastructural architecture. The nuclear envelope was intact, the nucleus preserved its oval morphology, and chromatin was uniformly dispersed with prominent euchromatic areas. The cytoplasm contained well-organized organelles, and no evidence of mitochondrial swelling, membrane disruption, or cytoplasmic vacuolization was observed. Perinuclear and intracellular spaces appeared normal, indicating preserved neuronal integrity (Figure 5A). In the cerebral IR group, neurons subjected to IR demonstrated pronounced ultrastructural degeneration. The nucleus showed irregular contours and condensed, clumped heterochromatin, consistent with ischemia-related nuclear pyknosis. Cytoplasmic edema and marked pericellular vacuolization were evident. Organelles were poorly defined, and mitochondrial swelling with disrupted cristae was frequently observed. These findings are characteristic of severe neuronal injury induced by cerebral IR (Figure 5B). In the IR+Dex group, neurons in the treatment group exhibited partially preserved ultrastructure compared with the IR group. Although mild chromatin condensation was present, the nuclear membrane remained largely intact. Cytoplasmic edema was reduced, and mitochondrial morphology appeared closer to normal, with less pronounced swelling and more preserved cristae. The decreased vacuolization and improved nuclear-cytoplasmic integrity suggest that the administered agent exerted a protective effect against cerebral IR-induced ultrastructural damage (Figure 5C).

### 3.8. Bioinformatic Network Analysis Identifies Key Dex-Responsive Apoptotic Pathways

**Figure 6:**
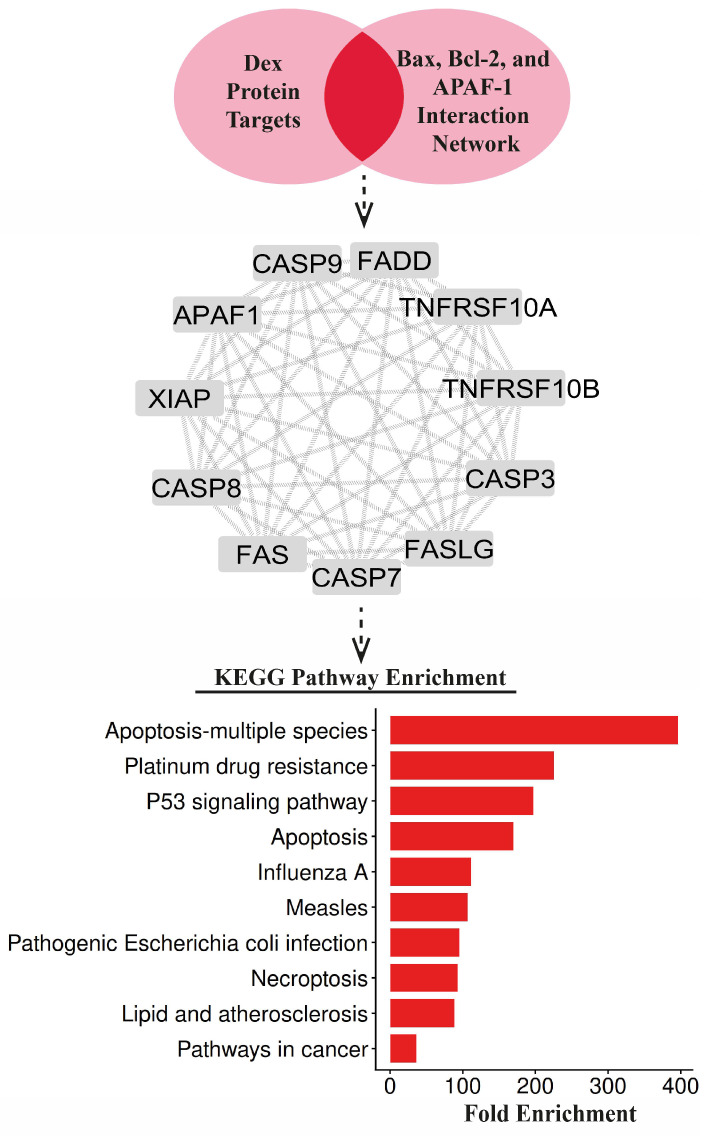
Integration of Dex targets with Bax, Bcl-2, and APAF-1 networks and KEGG pathway enrichment. Shared proteins between Dex targets and Bax, Bcl-2, and APAF-1 networks are shown with their interactions. The bar graph displays the top significant enriched KEGG pathways ranked by fold enrichment (FDR < 0.05).

The intersection of Dex target proteins with apoptosis-related regulators (Bax, Bcl-2, and APAF-1) yielded ten common proteins: Fas cell surface death receptor (FAS), Caspase (Casp) family members Casp3, Casp7, Casp8, and Casp9, Apoptotic peptidase activating factor-1 (APAF-1), Fas ligand (FASLG), Fas-associated death domain protein (FADD), Tumor necrosis factor receptor superfamily member 10A (TNFRSF10A), TNFRSF10B, and X-linked inhibitor of apoptosis protein (XIAP). Among the intersecting targets, APAF-1 emerged as a predicted Dex-associated protein within the intersecting network. Functional enrichment analysis of these intersecting proteins using the KEGG database identified statistically significant pathways (FDR < 0.05), ranked by fold enrichment. The apoptosis pathway was predominantly enriched, while p53 signaling and necroptosis pathways were also specifically identified among the significantly enriched results (Figure 6).

### 3.9. Molecular Docking Reveals Stable Binding of Dex to Core Apoptosis Regulators

Molecular docking analysis demonstrated that Dex interacts with multiple apoptosis-related proteins, including Bax, Bcl-2, APAF-1, Caspase-3, Caspase-8, and Caspase-9, with binding energies ranging between −6.1 and −8.7 kcal/mol (Table 3). Among these, Caspase-3 and Bcl-2 showed the most favorable interactions (≤−8 kcal/mol), indicating high binding stability. Dex bound to Bax primarily at the BH3-recognition groove, forming hydrophobic contacts with Leu63, Val83, and Gly108, consistent with known BH3-peptide interaction regions. For Bcl-2, the ligand occupied the canonical hydrophobic BH3 binding pocket, establishing stabilizing contacts with Phe101, Ala108, and Arg143, suggesting potential modulation of mitochondrial anti-apoptotic signaling. Docking to APAF-1 revealed a moderate-affinity interaction (−6.1 kcal/mol) localized near the nucleotide-binding domain, involving residues Thr163 and Lys192, indicating a possible influence on apoptosome assembly regulation. In Caspase-3, Dex fits into the catalytic cleft adjacent to the Cys163–His121 catalytic dyad, generating hydrogen bonds with Ser205 and hydrophobic contacts with Val266, yielding a strong predicted affinity (−8.4 kcal/mol). Similar interactions were observed for Caspase-8 and Caspase-9, where the ligand engaged residues surrounding the respective active sites (Casp8: His317, Cys360; Casp9: Cys287, His237), producing docking scores of −7.2 and −7.8 kcal/mol. Overall, the binding profiles suggest that Dex may exert anti-apoptotic effects not only through receptor-mediated signaling but also via predicted molecular interactions with multiple components of the intrinsic and extrinsic apoptotic machinery. These findings complement the immunohistochemical evidence of reduced Bax expression and preserved Bcl-2 immunoreactivity in treated groups.

### 3.10. Molecular Docking Reveals Association Between Dex and APAF-1

**Figure 7:**
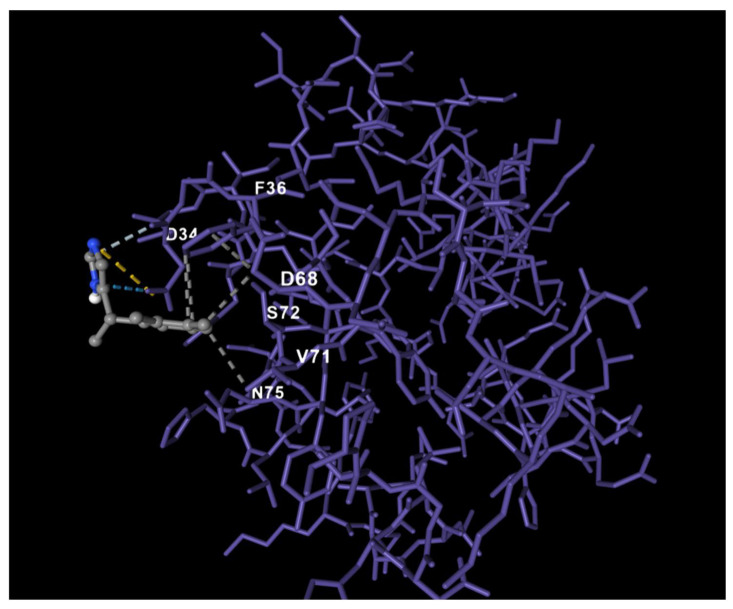
Predicted molecular docking interaction between dexmedetomidine and APAF-1. Dexmedetomidine is shown in ball-and-stick representation interacting with a surface pocket of APAF-1 (wire representation). The model illustrates a predicted binding pose based on docking analysis.

Molecular docking analysis was performed to explore the predicted interaction between Dex and APAF-1. In the best-scoring pose, Dex was accommodated at a surface pocket formed by the N-terminal region of APAF-1, involving residues F36, D34, D68, V71, S72, and N75. The ligand was oriented such that its polar moiety faced the charged side chains of D34 and D68, generating predicted hydrogen bonds/salt-bridge contacts (yellow and blue dashed lines). Additional stabilizing hydrogen bonding was observed with S72 and N75, while F36 and V71 contributed to a hydrophobic microenvironment around the aromatic ring of Dex. These multiple polar and hydrophobic contacts yielded a favorable docking score, suggesting a stable interaction between Dex and the APAF-1 surface. Given that this region belongs to the N-terminal regulatory domain of APAF-1, which participates in apoptosome assembly and caspase-9 recruitment, Dex binding at this site may sterically or allosterically interfere with pro-apoptotic signaling, providing a structural explanation for the anti-apoptotic effects observed in our cerebral IR model (Figure 7).

## 4. Discussion

The present study demonstrates that Dex exerts partial but significant neuroprotective effects against cerebral IR injury in an experimental rat model. These findings are consistent with the growing body of evidence from myocardial, intestinal, and hepatic IR models, which collectively highlight Dex as a potent anti-inflammatory, anti-oxidative, and anti-apoptotic pharmacological agent [30,31,32].

In the current study, cerebral IR markedly disrupted systemic redox homeostasis, as reflected by decreased SOD, CAT, and GSH-Px activities and elevated MDA and TOS levels. These alterations are well-aligned with the oxidative surge reported in cardiac and hepatic IR models, in which excessive ROS generation during reperfusion overwhelms endogenous antioxidant defenses, leading to mitochondrial dysfunction and lipid peroxidation [30,33,34,35]. In line with these studies, Dex treatment in our model partially restored antioxidant enzyme activities while significantly reducing MDA levels, suggesting suppression of ROS-mediated cellular injury. Comparable antioxidant restoration following Dex administration has been demonstrated in hyperlipidemic myocardial IR rats [36], in hypoxia–reoxygenation hepatocytes, and in intestinal IR tissues, indicating a conserved and robust antioxidative mechanism [37].

Histopathological examination further supported these biochemical findings. Cerebral IR induced neuronal pyknosis, cytoplasmic eosinophilia, perivascular/perineuronal edema, and vacuolation (hallmark indicators of reperfusion injury). Similar structural degeneration has been extensively documented in Dex-responsive models involving the heart, intestine, and liver, where Dex mitigates vascular permeability, edema formation, and inflammatory infiltration [38,39]. Consistent with these reports, Dex markedly reduced the severity of neuronal degeneration in our study, highlighting its capacity to preserve cytoarchitectural integrity under ischemic stress.

The most striking mechanistic insight arose from our immunohistochemical evaluation of apoptotic markers. Cerebral IR markedly increased Bax and APAF-1 expression, both of which are central mediators of mitochondrial apoptosis. Increased Bax facilitates mitochondrial outer membrane permeabilization, whereas APAF-1 promotes apoptosome formation and caspase-9 activation. These findings parallel those from myocardial and hepatic IR models, where Dex was shown to reduce cleaved caspase-3, Bax, and cytochrome-c release while improving Bcl-2 expression [40,41]. In the present study, Dex significantly increased Bcl-2 and reduced Bax and APAF-1 expression, demonstrating that its anti-apoptotic effects are preserved in the central nervous system.

The use of female rats with estrous cycle monitoring was intended to minimize hormonal variability and enhance internal validity. Nevertheless, sex-related factors may influence ischemia/reperfusion injury and neuroprotective responses, as estrogen and progesterone are known to modulate oxidative stress, mitochondrial function, and apoptotic signaling. Therefore, while the present findings are robust within a hormonally controlled female cohort, extrapolation to male animals or mixed clinical populations should be approached with caution. Future studies incorporating both sexes may further clarify potential sex-dependent differences in dexmedetomidine-mediated neuroprotection.

The bioinformatic analysis closely aligned with the in vivo findings and further supported the regulatory effect of Dex on apoptotic signaling pathways. Identification of ten intersecting targets between Dex-associated proteins and the Bax, Bcl-2, and APAF-1 networks, including FAS, FADD, FASLG, XIAP, and multiple caspase family members, indicates that Dex exerts a multifaceted modulatory influence on both intrinsic and extrinsic apoptosis [42,43,44]. Notably, the emergence of APAF-1 as a predicted potential Dex target is highly consistent with the marked reduction in APAF-1 immunoreactivity observed in our experimental results [45].

KEGG enrichment analysis revealed statistically significant involvement of the apoptosis, p53 signaling, and necroptosis pathways [46]. These results strongly support the experimental evidence showing that Dex suppresses mitochondrial apoptosis induced by IR, characterized by increased Bax, decreased Bcl-2, and elevated APAF-1 expression. Enrichment of the p53 pathway highlights the central role of pro-apoptotic transcriptional activation after IR, whereas the reversal of Bax/Bcl-2 imbalance with Dex suggests functional regulation along this axis [31]. Additionally, identification of the necroptosis pathway indicates that IR triggers not only apoptosis but also regulated necrotic cell death. Considering previous reports demonstrating Dex-mediated suppression of the RIPK1–RIPK3–MLKL axis, modulation of necroptotic signaling in our model also appears plausible [44,47].

Molecular docking further supported these findings, demonstrating strong binding affinity of Dex to Casp3, Casp9, and Bcl-2 (≤−8 kcal/mol), and moderate affinity for APAF-1. Molecular docking analysis suggested potential structural interactions between Dex and apoptosis-related proteins, supporting but not proving a direct molecular effect. These computational results parallel the immunohistochemical observations, including reduced APAF-1, decreased Bax, and increased Bcl-2 expression. It should be emphasized that molecular docking and network analyses are predictive and hypothesis-generating in nature [48]. These in silico findings identify potential interaction sites and pathway associations but do not constitute direct experimental evidence of molecular binding. Nevertheless, they provide a rational framework for future validation studies, such as in vitro binding assays, functional inhibition experiments, or targeted modulation of apoptosis-related proteins.

Dexmedetomidine does not act as a classical free radical scavenger but exerts indirect antioxidant effects through modulation of sympathetic activity, mitochondrial function, and intracellular signaling pathways [46,49]. Previous studies have demonstrated dose-dependent effects of dexmedetomidine, with pro-apoptotic responses reported at supratherapeutic concentrations [45]. The dose used in the present study was selected to approximate clinically relevant exposure and falls within the neuroprotective range reported in the literature [50]. The present study focuses on acute dexmedetomidine exposure in an experimental ischemia/reperfusion model. While clinical observations suggest potential neuroprotective benefits during perioperative or intensive care use, the effects of long-term or chronic dexmedetomidine administration remain insufficiently characterized and warrant further investigation [51].

From a translational perspective, the timing of dexmedetomidine administration in this study was designed to reflect clinically relevant neuroprotective windows [52,53]. Pre-ischemic and early reperfusion exposure may model perioperative settings, such as major vascular or cardiac surgery, where dexmedetomidine is administered before and during ischemic risk periods [54,55]. Early post-ischemic exposure may also be relevant to acute stroke management scenarios in which neuroprotective agents are administered shortly after reperfusion [53]. Although experimental conditions cannot fully replicate clinical complexity, this administration schedule provides insight into plausible therapeutic windows for dexmedetomidine-mediated neuroprotection.

In summary, the present study demonstrates that dexmedetomidine confers neuroprotection in cerebral ischemia/reperfusion injury through coordinated modulation of oxidative stress, apoptotic signaling, and neuronal ultrastructure. The integrative design, combining in vivo biochemical and histopathological findings with immunohistochemical quantification and in silico analyses, represents a key strength of this work. These findings not only advance mechanistic understanding of dexmedetomidine-mediated neuroprotection but also support its potential translational relevance in ischemia-related brain injury, particularly in perioperative and acute reperfusion settings.

## 5. Conclusions

Within the limitations of an experimental cerebral IR rat model, these findings suggest that Dex may represent a promising neuroprotective strategy. Integrated histological, ultrastructural, and immunohistochemical assessments showed that Dex preserved neuronal integrity, while bioinformatic and molecular docking analyses supported its regulatory influence on key apoptosis-related pathways. These convergent findings indicate that Dex is a promising therapeutic candidate for mitigating ischemic neuronal damage, warranting further investigation in translational and clinical settings.

## Figures and Tables

**Table 1:** Mean ± Standard Deviations (SD) of serum biochemical values (SOD, CAT, GSH-Px, TAS, TOS, and MDA) for all groups.

Parameter	Control	IR	IR+Dex	*p*-Value
**SOD (ng/mL)**	24.57 ± 3.60	17.00 ± 3.45 †	20.75 ± 1.28 *	<0.05
**CAT (ng/mL)**	99.81 ± 4.32	88.30 ± 3.26 †	95.81 ± 2.24 *	<0.05
**GSH-Px (U/mL)**	119.48 ± 5.84	103.24 ± 5.54 †	110.74 ± 2.90 *	<0.05
**TAS (mmol Trolox equiv./L)**	1.80 ± 0.28	1.23 ± 0.31 †	1.40 ± 0.14 *	<0.05
**TOS (µmol H_2_O_2_ equiv./L)**	1028.03 ± 16.76	1205.05 ± 48.47 †	1136.75 ± 41.82 *	<0.05
**MDA (nmol/mL)**	0.67 ± 0.08	1.45 ± 0.36 †	0.98 ± 0.22 *	<0.05

Data are presented as mean ± SD. Statistical analysis was performed using one-way ANOVA followed by Tukey’s post hoc test. † *p* < 0.05 vs. control group. * *p* < 0.05 vs. IR group. IR: ischemia/reperfusion; Dex: dexmedetomidine; SOD: superoxide dismutase; CAT: catalase; GSH-Px: glutathione peroxidase; TAS: total antioxidant status; TOS: total oxidant status; MDA: malondialdehyde.

**Table 2:** QuPath-based quantitative analysis of Bax, Bcl-2 and APAF-1 immunostaining in brain tissue (mean ± SD).

Marker	Group	% Positive Cells (Mean ± SD)	H-Score (Mean ± SD)	*p*-Value (vs. IR)
**Bax**	Control	12.0 ± 3.5	45.0 ± 10.2	*p* < 0.001
	IR	48.5 ± 8.1	165.2 ± 20.4	
	IR+Dex	28.7 ± 5.6	98.3 ± 15.7	*p* < 0.001
**Bcl-2**	Control	52.3 ± 7.4	180.5 ± 22.1	*p* < 0.001
	IR	21.4 ± 5.0	75.6 ± 14.3	
	IR+Dex	40.1 ± 6.8	138.9 ± 18.5	*p* < 0.001
**APAF-1**	Control	10.5 ± 2.9	40.2 ± 9.7	*p* < 0.001
	IR	43.8 ± 7.5	158.7 ± 19.3	
	IR+Dex	24.9 ± 4.9	103.5 ± 16.4	*p* < 0.001

**Table 3:** Predicted binding energies (kcal/mol) of Dex with apoptosis-related proteins.

Protein	Binding Energy (kcal/mol)	Key Interacting Residues	Binding Pocket
**Bax**	−7.4	Leu63, Val83, Gly108	BH3-binding groove
**Bcl-2**	−8.1	Phe101, Ala108, Arg143	BH3 hydrophobic pocket
**APAF-1**	−6.1	Thr163, Lys192	Nucleotide-binding domain
**Caspase-3**	−8.4	His121, Cys163, Ser205	Catalytic cleft
**Caspase-8**	−7.2	His317, Cys360	Active site
**Caspase-9**	−7.8	Cys287, His237	Catalytic groove

## Data Availability

The original contributions presented in this study are included in the article. Further inquiries can be directed to the corresponding author.
